# Reasons to Engage in and Learning Experiences From Different Play Strategies in a Web-Based Serious Game on Delirium for Medical Students: Mixed Methods Design

**DOI:** 10.2196/18479

**Published:** 2020-07-29

**Authors:** Kiki R Buijs-Spanjers, Harianne HM Hegge, Fokie Cnossen, Debbie ADC Jaarsma, Sophia E de Rooij

**Affiliations:** 1 Department of Geriatric Medicine University of Groningen University Medical Center Groningen Groningen Netherlands; 2 Center for Education Development and Research in Health Professions, LEARN University of Groningen University Medical Center Groningen Groningen Netherlands; 3 Bernoulli Institute of Mathematics, Computer Science and Artificial Intelligence Department of Artificial Intelligence University of Groningen Groningen Netherlands; 4 Medical Spectrum Twente Medical School Twente Enschede Netherlands

**Keywords:** dark play, serious games, medical education, medical students, delirium

## Abstract

**Background:**

Although many studies have recently been published on the value of serious games for medical education, little attention has been given to the role of dark play (choosing unacceptable actions in games).

**Objective:**

This study aimed to investigate potential differences in the characteristics of medical students who have the opportunity to choose normal or dark play in a serious game. This study also aimed to compare their reasons for choosing a play strategy and their perceptions of what they learned from their game play.

**Methods:**

We asked undergraduate medical students to play a serious game in which they had to take care of a patient with delirium (The Delirium Experience). After getting acquainted with the game, students could opt for normal or dark play. Student characteristics (age, gender, experience with caring for older or delirious patients, and number of completed clerkships) were collected, and the Delirium Attitude Scale and Learning Motivation and Engagement Questionnaire were administered. Reasons for choosing normal or dark play were evaluated with an open-ended question. Information on lessons they had learned from the game was collected using an open-ended question and self-reported knowledge on delirium.

**Results:**

This study had 160 participants (89 normal play, 71 dark play). Male students (26/160, 56.5%) chose dark play significantly more often than female students (45/160, 39.5%; *P*=.049). We did not find significant differences in student characteristics or measurement outcomes between play strategies. Participants’ main reason for choosing normal play was to learn how to provide care to delirious patients, and the main reason for dark play was to gain insight into what a delirious patient has to endure during delirious episodes. All participants learned what to do when taking care of a delirious patient and gained insight into how a patient experiences delirium. We found no differences in self-reported knowledge.

**Conclusions:**

When medical students have the opportunity to choose dark play in a serious game, half of them will probably choose this play strategy. Male students will more likely opt for dark play than female students. Choice of play strategy is not affected by any other student characteristic or measurement outcome. All students learned the same lessons from playing the game, irrespective of their learning strategy.

## Introduction

Players can engage in serious games with different play strategies. However, little is known on potential differences in characteristics of players in these different play strategies, their motivation to engage in that play strategy, or what they learn from it. In this study, we used a serious game on delirium to investigate these knowledge gaps.

Providing care for delirious patients poses a great burden on health care professionals [1,2]. Delirium is an acute neuropsychiatric syndrome that is characterized by altered attention, awareness, and cognition. Delirium is associated with longer hospital stays, functional decline, institutionalization, and mortality [3]. For patients, delirium also has negative effects on their psychological and emotional wellbeing [4,5]. Hence, it is important to understand delirious patients’ needs when providing care to these patients, but, apparently, such understanding is often lacking [6,7]. Delirium often goes unrecognized by health professionals due to a lack of knowledge, awareness, and education about delirium. An overlap in symptoms of delirium and dementia makes it even harder to provide good quality care to delirious patients [1,8].

Current educational interventions mainly focus on knowledge and skills in recognizing delirium [9,10]. To improve delirium care, however, educational interventions need to have a different and broader focus. This includes gaining a better understanding of the patients’ needs and health care professionals’ attitudes towards delirious patients as well as promoting knowledge transfer to help learners develop knowledge of how to care for delirious patients [6,7]. It is important that educational interventions aimed at facilitating knowledge transfer encourage experiential learning in which learners are actively engaged with the study material [1]. In experiential learning, learners have to grasp and transform their experiences to create knowledge. In doing so, it is important that they are able to experiment with different approaches [11].

Serious games are interventions that promote experiential learning by providing a safe environment where learners can practice without the risk of harming the patient [12]. Serious games provide playful learning experiences that can be applied to real-life settings and actively involve learners [12]. They also give the learners autonomy on what they want to do, allowing them to experiment with different care options, which in turn will increase their feeling of control and satisfaction [13]. Moreover, serious games as experiential learning tools simultaneously allow learners to use different play strategies in the game (ie, normal or dark play). We define normal play strategy as choosing options in the game that resemble acceptable choices in real life. Another play strategy that players may opt for is dark play, during which players show in-game behaviors that are unacceptable in real life [14]. Experimenting with different types of care and options available in a serious game could provide learners with additional insights and knowledge [12,15].

Although many studies on serious games in medical education have been reported in recent years [16-18], these studies often investigated serious games as whole artifacts without focusing on the effect of different play strategies such as dark play. In a previous study, we showed that dark play did not affect game effectiveness [19]. However, our students had been allocated to a normal or dark play condition without being able to choose their game. Because of the allocation to a specific play strategy in the previous study, little is yet known about how often students voluntarily choose dark play in a serious game, which students choose to engage in dark play and why, and what they gain from their experience. By gaining insight on these aspects, we may meet the demand of more research on game elements that promote engagement and support the learning of players [16,18,20,21].

To be able to use dark play in serious games more efficiently in education and enhance learning by giving students an opportunity to experience the consequences of wrong choices and actions in a safe environment, more research on these topics is required. In this study, we sought to identify potential differences in characteristics between medical students who choose and do not choose to engage in dark play in a serious game on delirium. We examined their reasons for choosing normal or dark play and their perceptions of their learning experiences.

## Methods

### Educational Background

The master’s program in medicine of the University Medical Center Groningen (UMCG) consists of 3 years. The first year is a dual learning year with 4 blocks, where each block consists of 5-week just-in-time skills training in a skills lab setting followed by 5-week “junior” clerkships. The second year comprises a series of ten 4-week “senior” clerkships, and the third year consists of a 20-week clinical elective and a 20-week research elective. Every 6 weeks, approximately 20 first-year master’s students start their junior psychiatry clerkship. They play The Delirium Experience as part of their introductory program.

### Participants, Recruitment, and Ethical Considerations

Participants in this study were first-year students of the master’s program in medicine of the UMCG who were at the start of their psychiatry clerkship.

Between January 2018 and January 2019, at the start of each clerkship, all students were asked to participate in our study by the first author (KBS). They were informed about the purpose of the study, and afterwards they received digital information and a digital informed consent form. Participation was voluntary and could be stopped at any time. Students were also allowed to play the game without participating in the study. To ensure students did not feel obliged to participate, the researchers were not involved in other educational activities. All 160 students agreed to participate and signed the informed consent form.

Registration of the trial was not necessary in accordance with the International Committee of Medical Journal Editors recommendations.

### Intervention

**Figure 1:**
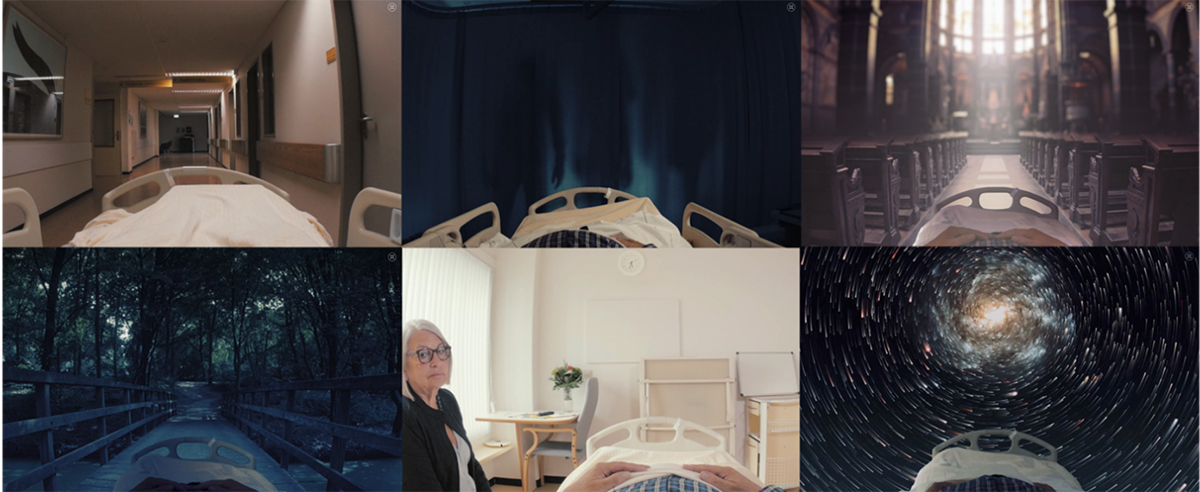
Screenshots of the Delirium Experience serious game, from the patient's perspective.

**Figure 2:**
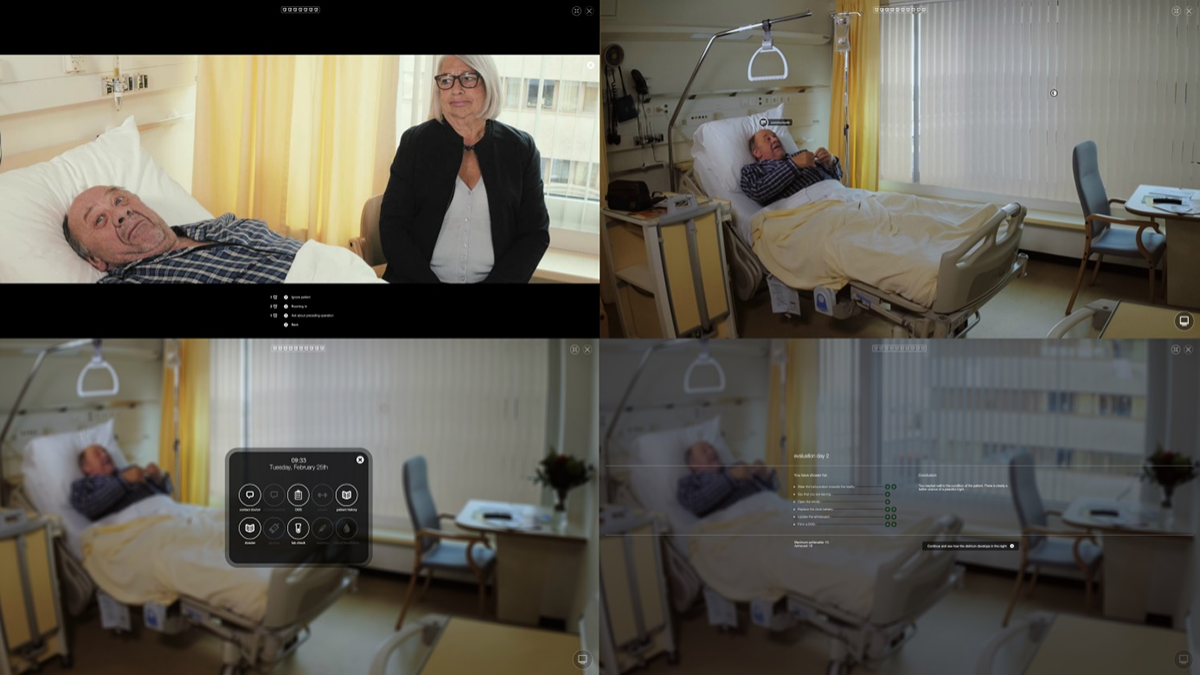
Screenshots of the Delirium Experience serious game, from the health care professional’s perspective.

In our research, we used The Delirium Experience (Multimedia Appendix 1), a desktop simulation-based serious game that forces players to explore two different perspectives on delirium: that of a delirious patient (see Figure 1 for screenshots) and that of a health care professional (see Figure 2 for screenshots). The Delirium Experience was specifically developed to provide players with insight into what a delirious patient has to endure and how their actions and decisions as health care professionals may affect delirious patients and their interests [22].

The game works like this: during the daytime for 4 consecutive days, players take the role of a health care professional who provides care to a delirious patient. During the 4 nights, they switch to the patient’s perspective to experience being delirious. Depending on the actions they choose during the day, their story will play out quite differently, and the delirious episodes will develop differently during the night. If players make the right care choices and provide good care, delirious episodes will be less severe than if they had made the wrong choices. Accordingly, the Delirium Experience enables players to opt for dark play by choosing wrong actions as a health professional and making the delirious episodes as severe as possible. Completing the entire game (all 4 days) takes about 20 minutes.

Players receive both direct and indirect feedback from the game. At the end of each day as a health care professional, they receive feedback on the consequences of their actions for the severity of the delirium and an overview of how their actions affected delirium severity. During the nights, players receive indirect feedback by experiencing the patient’s responses to the actions of the health care professional during the delirious episodes.

### Study Design and Procedure

We used triangulation during the interpretation of our findings to converge both quantitative and qualitative outcomes in a mixed-method design. We therefore concurrently gathered quantitative and qualitative data but analyzed these datasets separately.

Before playing The Delirium Experience, participants were asked to answer background questions about their age, gender, whether they had experience with caring for older and delirious patients (yes/no), and the number of clerkships they had completed. We also asked them to self-report their knowledge of delirium and to complete the Delirium Attitude Scale.

Participants played The Delirium Experience twice. The first time was meant to gain familiarity with the game. Before playing it a second time, students received a written explanation of the normal and dark play options in the game. When they played the game again, they had the opportunity to opt for another game play. They had the choice between playing the game with the intention to provide the best possible care to the patient (normal play; ie, guiding the patient through his hallucinations and/or supporting orientation) or to make the symptoms of delirium as severe as possible with their actions as a health care professional (dark play; ie, denying the patient’s hallucinations and/or using sedation).

After playing, participants completed the Motivation and Engagement Questionnaire to evaluate their learning experiences. In addition, we asked them to indicate whether they had opted for normal or dark play, their reasons for choosing either normal or dark play, and what they had learned from it. Once again, we asked the participants to self-report their knowledge on delirium.

### Outcome Measures

Self-reported knowledge on delirium was measured on a scale from 0 to 10, with higher scores indicating higher levels of perceived knowledge. In the Netherlands, it is common to use a mark like this that indicates your knowledge level. Marks ≥5.5 are considered sufficient knowledge, where marks <5.5 represent insufficient levels of knowledge.

Participants’ attitudes towards delirious patients were measured using the Delirium Attitude Scale. This scale consists of 19 items that are scored on a 7-point Likert scale, with scores ranging from 19 to 133 points. Higher scores reflect a more positive attitude [23].

Participants’ learning experiences were evaluated with the Motivation and Engagement Questionnaire, consisting of 9 items that are scored on a 5-point Likert scale, with scores ranging from 9 to 45 points [24]. Higher scores reflect more participant motivation and engagement in learning.

To uncover the reasons why participants chose normal or dark play, we added an open-ended question at the end of the digital questionnaire: “Why did you choose to play The Delirium Experience in normal or dark play?”

To investigate what participants had learned from their experience, we measured self-reported knowledge on delirium again (range 0-10) and asked the open-ended question: “What new insights did you gain while playing The Delirium Experience for the second time?”

### Data Analysis

#### Quantitative Analysis

We checked data for normality by judging histograms, skewness, and kurtosis. To test for differences between participants who chose to engage in normal or dark play, we analyzed discrete variables (gender, experience with caring for older and delirious patients, and the number of clerkships they had completed) using chi-square tests and continuous variables (age, self-reported knowledge, attitude, and learning motivation and engagement) using independent samples *t* tests. *P* values <.05 were considered statistically significant. These statistical tests were performed with SPSS 23.0.

#### Qualitative Analysis

We thematically analyzed answers to the two open-ended questions (“Why did you choose to play The Delirium Experience in normal or dark play?” and “What new insights did you gain while playing The Delirium Experience for the second time?”) with Atlas.ti software, version 8 (ATLAS.ti Scientific Software Development GmbH, Berlin, Germany). We created separate data files to collect and analyze the responses to each open-ended question. During the coding process, the researchers were blinded to the participants’ play strategy to ensure objectivity. After coding, we added information on the participants’ play strategy to the data in order to analyze the differences.

We used inductive content analysis with constant comparison to find similarities and differences in students’ answers between those who chose normal or dark play [25,26]. The whole process of qualitative data analysis was as follows. At first, the first author (KBS) read all answers to the open-ended questions to become familiar with the data. Subsequently, we identified initial codes, and KBS started coding the entire dataset. The resulting framework was iteratively refined as new, inductive codes were generated and integrated. Next, we identified preliminary themes by grouping similar concepts. Two researchers (KBS and DJ) reviewed and refined these preliminary themes to generate final themes. We analyzed the data in their original language; the most illustrative quotes were translated into English.

## Results

### Overview

In this section, we first describe the results of the baseline questions and reported learning motivation and engagement after playing The Delirium Experience, separately for students who chose normal or dark play. In the second part of this section, we describe students’ self-reported reasons to engage in normal or dark play, and lastly, we report the results for students’ self-reported knowledge gain in normal or dark play.

### Participants Who Chose Normal or Dark Play

In total, 160 students participated in this study; 89 (56%) chose to play The Delirium Experience in normal play, and 71 (44%) chose dark play. Our study population consisted of 46 (46/160, 29%) male and 114 (114/160, 71%) female students, which is representative of the general student population of the master’s program in medicine of the UMCG. Participants who chose normal or dark play did not significantly differ in age, experience with caring for older or delirious patients, or number of completed clerkships (Table 1). However, we found that male participants chose dark play significantly more often than female participants (56.5% [26/46] of men vs 39.5% [45/114] of women; *P*<.049). We did not find significant differences in self-reported knowledge on delirium, attitudes towards delirious patients before playing, or learning motivation and engagement after playing (Table 2).

**Table 1:** Characteristics of participants who chose normal or dark play.

Characteristics		Total (n=160)	Normal play (n=89)	Dark play (n=71)	*P* value
Age (years), mean (SD)^a^		23.0 (2.6)	23.6 (2.9)	23.1 (2.1)	.31
**Gender, n (%)^b^**					.049
	Male	46 (28.8)	20 (43.5)	26 (56.5)	
	Female	114 (71.2)	69 (60.5)	45 (39.5)	
**Experience with older patients, n (%)^b^**					.70
	Yes	104 (65.0)	59 (56.7)	45 (43.3)	
	No	56 (35.0)	30 (53.6)	26 (46.4)	
**Experience with delirious patients, n (%)^b^**					.78
	Yes	56 (35.0)	32 (57.1)	24 (42.9)	
	No	104 (65.0)	57 (54.8)	47 (45.2)	
**Number of completed clerkships, n (%)^b^**					.39
	0	39 (24.4)	19 (48.7)	20 (51.3)	
	1	38 (23.8)	23 (60.5)	15 (39.5)	
	2	52 (32.5)	27 (51.9)	25 (48.1)	
	3	28 (17.5)	17 (60.7)	11 (39.3)	
	≥4	3 (1.9)	3 (100)	0 (0.0)	

^a^Data compared using independent samples *t* tests.

^b^Data compared using chi-square tests.

**Table 2:** Mean scores of self-reported knowledge, attitudes, and learning motivation and engagement in participants who chose normal or dark play.

Characteristic	Total (n=160)	Normal play (n=89)	Dark play (n=71)	*P* value^a^
Self-reported knowledge (possible score range, 0-10), mean (SD)	5.1 (1.9)	4.9 (1.9)	5.3 (1.8)	.23
Attitude (possible score range, 19-133), mean (SD)	90.8 (10.7)	91.1 (11.4)	90.4 (9.8)	.66
Learning motivation and engagement (possible score range, 9-45), mean (SD)	35.0 (4.0)	35.3 (4.2)	34.6 (3.9)	.35

^a^Data compared using independent samples *t* tests.

### Reasons for Choosing Normal or Dark Play

Participants’ reasons for choosing normal or dark play could be divided into three main themes: (1) to learn about delirium (care), (2) students’ performance in the normal or dark game play, and (3) to take full advantage of the opportunities offered by the game.

#### To Learn About Delirium (Care)

A reason for participants to engage in normal play was that they considered learning how to provide good care for a delirious patient the most important and normal thing to do. One of the participants answered: “Because I feel it is more important [for me] to know how to act well.” On the other hand, participants who had chosen dark play wanted to gain insight into what a delirious patient has to endure during delirious episodes: “I wanted to experience – from the patient’s perspective – what it would be like to go through episodes of delirium.”

Furthermore, participants were interested in seeing the progression of delirium. Participants who chose normal play wanted to see how delirium develops when providing good quality care and gain insight into factors that decrease the severity of delirious episodes. Participants who had chosen dark play, on the contrary, wanted to see how severe delirious episodes develop and which factors influence this.

#### Students’ Performance in the Normal or Dark Game Play

A reason to choose either normal or dark play was that participants wanted to have a different game experience than they had in their first game. They thought it would be more instructive to see the effects of either correct or incorrect choices. For example, to explain why she used normal play, a participant answered: “During my first game play, I did not receive many points, and the delirium was quite severe, so I also wanted to see how delirium would progress if better treatment was given to the patient.” A participant who had chosen dark play answered: “During my first game play, I became aware of what I could have done better. Therefore, I thought it would be more instructive to see [the stages of] progression of severe delirium.”

In addition, participants who had chosen normal play wanted to see whether they had learned something from their first game play. They wanted to apply the knowledge they had obtained during their first game play and try to provide better care to the patient.

#### To Take Full Advantage of the Opportunities Offered by the Game

Another theme was the game itself and what its environment had to offer. In particular, participants who chose dark play indicated that the opportunities offered by the game environment was their reason to choose dark play: “In dark play, you can see what happens to a patient if you don’t take good care of him; in real life, you just want to treat the patient as well as possible and [be able to] recognize the signs of poor treatment.” These participants were also driven by curiosity about other scenarios in the game; as one participant said: “In the closing video, I saw some scenes with a doctor that I hadn’t seen in the game yet.”

### Lessons Learned From Playing The Delirium Experience in Normal or Dark Play

To study lessons participants had learned from normal or dark play, we measured their self-reported knowledge on delirium and asked an open-ended question on what they had learned after playing. We did not find any differences in self-reported knowledge on delirium between participants who had chosen normal or dark play (mean 6.8, SD 1.2 vs mean 6.7, SD 1.2; t_155_=0.361, *P*=.72).

Lessons participants had learned by playing The Delirium Experience for the second time can be divided into two themes: (1) an understanding of how to provide care to a delirious patient and (2) an understanding of the patient’s experience. There were also participants who stated that they gained no new insights after playing The Delirium Experience for a second time.

#### An Understanding of How to Provide Care to a Delirious Patient

Participants’ answers mainly focused on practical aspects of providing care to a delirious patient. First, participants saw the importance of guiding the patient, as stated by a participant who had chosen dark play: “The importance of good and correct contact with the patient, even though it seems hard due to the completely distracted [state of mind the] patient [was in].” Second, the importance of good orientation for the patient was frequently mentioned: “I experienced the game as really instructive and realized that small things, such as writing down the date and location, and opening the blinds, can contribute to decreased patient confusion.” Third, participants gained new insights into prescribing medication for delirium: “I also need to realize that giving medication is not the most important thing to do.”

Furthermore, participants gained more insights into how their actions as health care professionals could influence the patient and delirium: “As a health care professional, you are in control of how delirium develops, and you are able to worsen or improve it.”

Finally, participants who had chosen normal as well as participants who had chosen dark play reflected on their knowledge while playing the game. A participant who had chosen dark play answered: “I thought I already had quite some knowledge of how to handle older people with delirium, but when the game forces you to make choices, this knowledge seemed to be limited.”

#### An Understanding of the Patient’s Experience

Participants who had chosen normal play as well as participants who had chosen dark play gained new insights into how a patient experiences delirious episodes. Participants answered:

I’ve never realized what it would be like to experience it as a patient, so this [playing The Delirium Experience] was really clarifying.

The first time [I played The Delirium Experience], the delirium was not that exciting, but it was really scary for the patient the second time. It is good to see how frightening it can be.

Furthermore, participants who chose dark play mentioned they had not expected that delirious episodes would be that intense.

#### No Added Value

Some of the participants who had chosen normal or dark play answered that there was not much added value in playing The Delirium Experience twice. The main explanations were that the game lacked feedback with reasons why they should act in a certain way and that the first game play was already instructive enough. For example, participants answered:

Little. I missed an explanation on why certain decisions were either positive or negative.

Not really, the first time was more instructive.

## Discussion

### Study Aims

With this study, we investigated potential differences in medical students who could choose between normal and dark play of a serious game and their perceived learning experiences. We therefore compared characteristics of students who opted for normal or dark play in a serious game on delirium. We investigated why students chose normal or dark play and what lessons they learned regarding normal or dark play.

### Principal Findings

We found that male participants were more likely to choose dark play than female participants. We did not find any further differences between participants who chose normal or dark play in other characteristics (ie, age, experience with caring for older or delirious patients, and number of completed clerkships), attitude towards delirium, self-reported knowledge on delirium, and learning motivation and engagement. We grouped participants’ reasons for choosing normal or dark play into three themes: (1) to learn about delirium (care), (2) students’ performance in the normal or dark play game, and (3) to take full advantage of the opportunities offered by the game. The lessons participants learned after playing normal or dark play could be divided into two themes: (1) an understanding of how to provide care to a delirious patient and (2) an understanding of the patient’s experience. We did not find any differences in self-reported knowledge after playing normal or dark play.

Our finding that male participants chose dark play more often than female participants may be explained by the following: In entertainment games, men are often player types who are more interested in exploring the game environment [27]. The results of our study suggest this may also be the case in serious games. Furthermore, there is evidence that men and women tend to have different task orientations. For example, women may be more interested in normal play because they tend to be more mastery-oriented than men, who tend to be more performance-oriented [28]. Interestingly, participants who chose normal play indicated that (one of) their reasons for choosing this type of game play was to learn how to provide care to delirious patients. Additionally, since women prefer entertainment games in which they have to make meaningful decisions [27,29], in serious games they may also be more interested in choosing a play strategy that includes making meaningful decisions (ie, providing good care). Lastly, because female medical students tend to score higher on empathy than their male peers [30], it may be harder for them to choose unacceptable gaming options that would harm a patient in real life.

The results of our study indicate that students learn the same lessons, irrespective of their learning strategy. Participants’ reasons for choosing normal play centered around wanting to learn how to provide good quality care to delirious patients, while participants’ reasons for choosing dark play pertained to the opportunity to experience what a delirious patient has to endure. Both groups of participants learned how to provide care to a delirious patient and gained insight into how a patient experiences delirious episodes. Although all participants played the game using different strategies and consequently experienced different simulations, they all seem to have learned the same lessons. A disadvantage of simulation-based education, however, may be the variety of situations that can occur in a simulation, which may result in different experiences and knowledge after the simulation [31]. Yet, our results imply that engaging in different simulation situations in The Delirium Experience (eg, the severity of the delirious episodes) does not result in differences in lessons learned or self-reported knowledge. This is in line with the results of our previous study showing that normal or dark play did not affect game effectiveness in students who were allocated to the two conditions [19].

Other authors have previously advocated that educational interventions on delirium should focus more on transfer of knowledge to practice [6,10]. Although we did not investigate providing care to a delirious patient in actual practice, participants did report many practical actions that can also help improve delirium care in practice. In addition, the aim of the Dutch Delirium Guidelines for health care professionals is to improve early recognition and treatment of delirium and delirium care [32]. It is therefore important that medical students are aware of the recommendations outlined in these guidelines to be able to provide good-quality delirium care. Many of the lessons our participants perceived to have learned during their game play were in line with the recommendations in the Dutch Delirium Guidelines (eg, guiding the patient and facilitating patient orientation).

### Further Research

The feedback in the game was provided in two ways: directly at the end of each day in the game itself and in the form of the patient’s response to the care choices made. Some participants indicated that playing the game using normal or dark play did not provide them with new insights, because they felt that feedback was lacking or unclear. One of the barriers for using feedback effectively may be students’ inability to decode feedback [33]. Effects of feedback are strong when the feedback message is encouraging and specific [34]. To improve the effect of feedback in serious games, it may be interesting to study how players who claimed to have learned nothing new from playing the game received and recognized feedback during their game play. Players who do not recognize or understand the feedback will not be able to benefit from it.

Our study showed that students gained more insight into what a delirious patient endures during delirious episodes; however, in actual practice, understanding of the patient’s needs is often lacking [6,7]. It would be interesting and relevant to study if and how students and health care professionals who work with delirious patients change their behaviors and attitudes when they encounter delirious patients in real life, after playing the game, particularly since destigmatization seems to occur when working closely with delirious patients who use to be stigmatized [35].

The demonstrated differences in female and male participants who chose normal or dark play are in line with the way male and female entertainment game players are categorized into player types [27,29]. However, research on gamification showed that design features can influence the preferences of player types [36]. Also, personality types and traits seem to play a role in which design features are most effective in serious games and gamification [37,38], which warrants further investigation into including personality traits when designing serious games for medical education. To develop tailored and effective serious games that match players’ preferences, further research could be performed on specific preferences of serious games players, especially since disliked game elements can negatively affect outcomes and participation [36].

### Conclusions

Serious games offer a safe environment for practicing real-life situations and for exploring options that are unacceptable in real life. Both types of game play can lead to the same learning outcomes. When students have an opportunity to play a serious game in dark play, almost half of the students will choose this type of game play. Male students are more likely to opt for dark play than their female peers. No other student characteristics influenced their choice of normal or dark play, nor did attitude, self-reported knowledge, or learning motivation and engagement. Irrespective of the strategy chosen, students reported the same lessons learned after playing a serious game on delirium in normal or dark play. They learned how to provide care to a delirious patient and gained insight into what a delirious patient endures.

## Appendix

Multimedia Appendix 1 Trailer of The Delirium Experience.

## Abbreviations

UMCG: University Medical Center Groningen.
